# Anti-cancer Activity of *Centipeda minima* Extract in Triple Negative Breast Cancer via Inhibition of AKT, NF-κB, and STAT3 Signaling Pathways

**DOI:** 10.3389/fonc.2020.00491

**Published:** 2020-04-09

**Authors:** Magnolia Muk-Lan Lee, Brandon Dow Chan, Wing-Yan Wong, Zhao Qu, Man-Sum Chan, Tsz-Wing Leung, Yushan Lin, Daniel Kam-Wah Mok, Sibao Chen, William Chi-Shing Tai

**Affiliations:** ^1^Department of Applied Biology and Chemical Technology, The Hong Kong Polytechnic University, Hong Kong, China; ^2^State Key Laboratory of Chinese Medicine and Molecular Pharmacology (Incubation), The Hong Kong Polytechnic University Shenzhen Research Institute, Shenzhen, China; ^3^Institute of Medicinal Plant Development, Chinese Academy of Medical Sciences and Peking Union Medical College, Beijing, China

**Keywords:** *Centipeda minima*, natural compounds, breast cancer, triple negative breast cancer (TNBC), anti-cancer

## Abstract

Breast cancer is the most commonly diagnosed cancer in females worldwide. Estimates from the World Health Organization (WHO) International Agency for Research on Cancer, suggest that globally, there were around 2.1 million new breast cancer cases and 627,000 deaths due to breast cancer in 2018. Among the subtypes of breast cancer, triple negative breast cancer (TNBC) is the most aggressive and carries the poorest prognosis, largest recurrence, and lowest survival rate. Major treatment options for TNBC patients are mainly constrained to chemotherapy, which can be accompanied by severe side effects. Therefore, development of novel and effective anti-cancer drugs for the treatment of TNBC are urgently required. *Centipeda minima* is a well-known traditional Chinese herbal medicine that has historically been used to treat rhinitis, sinusitis, relieve pain, and reduce swelling. Recent studies have shown that *Centipeda minima* exhibited efficacy against certain cancers, however, to date, no studies have been conducted on its effects in breast cancer. Here, we aimed to investigate the anti-cancer activity of the total extract of *Centipeda minima* (CME), and its underlying mechanism, in TNBC. In MDA-MB-231, we found that CME could significantly reduce cell viability and proliferation, induce apoptosis and inhibit cancer cell migration and invasion, in a dose and time-dependent manner. We showed that CME may potentially act via inhibition of multiple signaling pathways, including the EGFR, PI3K/AKT/mTOR, NF-κB, and STAT3 pathways. Treatment with CME also led to *in vitro* downregulation of MMP-9 activity and inhibition of metastasis. Further, we demonstrated that CME could significantly reduce tumor burden in MDA-MB-231 xenograft mice, without any appreciable side effects. Based on our findings, CME is a promising candidate for development as a therapeutic with high efficacy against TNBC.

## Introduction

Breast cancer is the most commonly diagnosed cancer and primary cause of cancer death in females. Estimates from the World Health Organization International (WHO) Agency for Research on Cancer, suggest that in 2018 there were around 2.1 million new breast cancer cases and 627,000 deaths due to breast cancer, worldwide (1). Breast cancer is a heterogenous disease, and can be divided into several subtypes based on receptor status and molecular classes. Triple negative breast cancer (TNBC) accounts for 5–25% of all breast cancers, and is classified as tumors that are clinically negative for estrogen receptor (ER), progesterone receptor (PR), and human epidermal growth factor receptor 2 [HER2; (2, 3)]. Compared to other types of breast cancer, TNBC is highly invasive and metastatic, and rapidly progressive. TNBC has a high rate of recurrence, poor prognosis, low survival rate, and is more common among young and pre-menopausal women (3–5). The above factors have led to increasing levels of attention amongst clinicians and scientists, and as such, TNBC has become a major topic in breast cancer research.

The ER, PR, and HER2 receptors all serve as molecular targets for therapeutic agents, however, the lack of these receptors in TNBC limits available treatment options mainly to cytotoxic chemotherapy-centered approaches (6). Though chemotherapy has been shown to be relatively effective in early stage TNBC, more than 30% of TNBC patients develop metastatic disease, and for these patients, median overall survival is only 13–18 months (7, 8). In addition, patients treated with chemotherapy suffer from numerous side effects, including hair loss, headaches and muscle pains, fatigue, nausea, vomiting, loss of appetite, and weight loss. Thus, there is a pressing need for novel alternative approaches with improved efficacy and reduced toxicity for the treatment of TNBC.

Herbal medicines, especially traditional Chinese medicine (TCM), have been commonly used in the prevention and treatment of disease for thousands of years. They are considered as natural therapies, with advantages including potent efficacy, minimal-to-no side effects, affordability, and a wide and abundant variety. Over the past several decades, the worldwide populace has become more receptive and welcoming to the use of TCM as a complementary or alternative medicine, and natural products have been viewed as a source of potential compounds in the search for novel drugs (9, 10).

*Centipeda minima* (L.) A. Braun & Asch. (CM), also known in Chinese as Ebushicao, is an annual herbaceous plant of Asteraceae family widely distributed in China and eastern tropical Asia. The whole plant has been employed as a TCM for millennia and is commonly treatment for indications such as rhinitis, sinusitis, cough, headache, swelling, and allergy (11). Phytochemical research has demonstrated CM mainly contain phenolic acids (e.g., isochlorogenic acid A and isochlorogenic acid C), flavonoids, and sesquiterpene lactones [e.g., Brevilin A, Arnicolide D; (12–16)]. Reports suggest that *Centipeda minima* extracts (CME) possess anti-inflammatory (17), anti-bacterial (18, 19), and anti-allergic (20) effects. Previous studies have demonstrated the anti-cancer effects of CME, and compounds extracted from CM, in various cancers including nasopharyngeal carcinoma (13, 14, 21, 22), leukemia (23), glioblastoma (24), lung cancer (25), and colon cancer (26), however, the effects of CME on breast cancer have to date, not been demonstrated.

In this study, we investigated the anti-cancer effects and molecular mechanisms of CME in TNBC. We provide the first evidence of the anti-TNBC effect of CME, and its potential for further study and development as a treatment for TNBC.

## Materials and Methods

### Plant Materials and Sample Preparation

As previously described (22, 27), *Centipeda minima* was collected in August 2010 in Xiangfan, Hubei Province, China (latitude, 32°04′ N; longitude, 112°05′ E), and authenticated by Prof. Sibao Chen based on morphological features. A voucher specimen (EBSC-016-09) was deposited at the herbarium of the State Key Laboratory of Chinese Medicine and Molecular Pharmacology, Department of Applied Biology and Chemical Technology, The Hong Kong Polytechnic University. The whole plant was air-dried and ground to coarse powder. Extraction procedures were conducted at room temperature. 0.3 g powdered sample was weighed in a 50 mL centrifuge tube and sonicated (540 W) at room temperature with 10 mL, 50% ethanol for 30 min. The mixture was then centrifuged at ~3,000 g for 5 min and then filtrated into a 25 mL volumetric flask. The extraction procedure was then repeated once more. Afterward, the residue was washed with 3 mL, 50% ethanol, and the solution was transferred to a volumetric flask. Finally, the sample solution was marked up to 25 mL and was filtrated through a 0.45 μM syringe filter before high performance liquid chromatography (HPLC) analysis. The authentication and chemical profiling of CME was conducted according to Chan et al. (27). In brief, the content of CME was determined by high performance liquid chromatography coupled with quadrupole time-of-flight mass spectrometry (HPLC-QTOF-MS) and high-performance liquid chromatography-diode array detection (HPLC-DAD) in our laboratory. A HPLC profile was generated to establish the most common constituents of CME, then, using HPLC-DAD, each batch of CME was compared to a HPLC profile established with 10 common components for quality control.

For all *in vitro* assays, CME was prepared in dimethyl sulfoxide (DMSO; Sigma Chemical Co., St. Louis, MO, USA) at a stock concentration of 100 mg/ml and stored at −80°C until use. It was diluted in culture medium for cell culture studies. For *in vivo* studies, CME was dissolved in 0.5% carboxymethylcellulose (CMC; Sigma Chemical Co., St. Louis, MO, USA) solution for oral administration to mice.

### Cell Lines and Reagents

MDA-MB-231 and MCF-7 human breast cancer cells were purchased from the American Type Culture Collection (Manassas, USA). All cells were maintained in Dulbecco's Modified Eagle Medium (DMEM; Life Technologies, USA) supplemented with 10% heat-inactivated fetal bovine serum (FBS) and penicillin/streptomycin (50 U/mL), at 37°C and 5% CO_2_. Cell lines were tested and confirmed to be free of mycoplasma contamination.

### Cell Viability

The 3-(4,5-Dimethylthiazol-2-yl)-2,5-diphenyltetrazolium bromide (MTT) assay was used to determine cell viability of MDA-MB-231 and MCF-7 cancer cells treated with CME. Cells were seeded in a 96-well-plate at a density of 5 × 10^3^ cells/well. After 24 h, cells were treated with various concentrations of CME for another 24, 48, or 72 h. Cells were then treated with 0.5 mg/mL MTT (Sigma, St. Louis, MO, USA) at 37°C for 4 h. Media was removed after incubation, and DMSO (Duksan, Korea) was added to each well. Absorbance at 570 nm in each well was measured using a Clariostar Monochromator Microplate Reader (BMG LABTECH, Germany). Three independent experiments were carried out. The IC_50_ of CME in the cell lines were calculated using Prism 7 (GraphPad Software, CA, USA).

### Cell Proliferation Assay

6 × 10^5^ MDA-MB-231 cells were seeded in 60 mm plates for 24 h. Cells were then treated with various concentrations (0, 2.5, 5, 10, or 20 μg/ml) of CME for 24, 48, and 72 h. The number of viable cells at each time point were counted with a cell counting hemocytometer using the trypan blue exclusion method, according to the manufacturer's protocol (Thermo Fisher Scientific, USA). Three independent experiments were carried out.

### Colony Formation Assay

MDA-MB-231 cells were seeded in triplicate at a density of 1 × 10^3^ cells per well in six-well plates. After 24 h incubation, cells were treated with various concentrations (0, 0.31, 0.63, 1.25, 2.5, or 5 μg/ml) of CME for 14-days. On day 7, cells were refreshed with medium containing the specific concentrations of CME. At the end of the experiment, cells were fixed with 4% paraformaldehyde for 4 h, stained with 2% Giemsa blue solution overnight, and rinsed with MilliQ water before imaging for quantification. Colony counts were quantified using ImageJ software.

### AnnexinV-7-AAD/PE Double Staining Assay

6 × 10^5^ MDA-MB-231 cells were incubated in 60 mm culture plates for 24 h. Cells were then treated with various concentrations (0, 2.5, 5, 10, or 20 μg/ml) of CME for another 24 or 48 h. Cells were harvested and suspended in annexin-binding buffer, and apoptosis levels were determined via flow cytometry using a BD Accuri C6 Flow Cytometer (BD, San Jose, CA, USA) and the PE Annexin V Apoptosis Detection Kit (BD), according to manufacturer's instructions.

### Wound Healing Assay

MDA-MB-231 cells were examined for their mobility via wound healing assay using ibidi culture inserts (ibidi, Gräfelfing, Germany), according to the manufacturer's instructions. MDA-MB-231 cells were seeded at a concentration of 4 × 10^4^ cells per compartment of the culture inserts, overnight. Inserts were then removed to create a wound, and cells were treated with different concentrations of CME (0, 2.5, 5, 7.5, or 10 μg/ml) for 48 h. Images were captured at 0, 4, 10, 24, and 48 h using an imaging microscope. The images were analyzed using the Automated Cellular Analysis System (ACAS; ibidi) to assess the migration characteristics of the cultured cells. The speed of wound closure was determined by the area of cell coverage against time.

### Invasion Assay

The invasiveness of MDA-MB-231 cells were examined using an invasion assay with BD BioCoat Growth Factor Reduced Matrigel invasion chambers (BD Biosciences, San Jose, CA, USA), according to the manufacturer's instructions. MDA-MB-231 cells were seeded in serum free medium in the upper chambers, at a density of 8 × 10^4^ cells/chamber. Complete medium was placed in the lower chamber. After 48 h incubation at 37°C, the remaining cells on the upper surface of the filter were removed using a cotton swab. Cells that invaded to the lower surface of the filter were fixed with methanol, stained with 2% Giemsa blue solution, rinsed with MilliQ water and counted under a light microscope (three fields, 100× magnification). Figures obtained were calculated by averaging the total number of cells from three fields. Experiments were conducted in triplicate.

### qPCR Assessment

The expression of MMP-9 in MDA-MB-231 cells was determined by quantitative RT-PCR. Total RNA was isolated from cells and colon samples using the E.Z.N.A.® Total RNA Kit I (Omega Bio-tek, USA), and quantified using a Nanodrop One spectrophotometer (Thermo Scientific, USA). First strand cDNA synthesis was carried out from 1 μg RNA using SuperScript® VILO™ MasterMix (ThermoFisher Scientific). PCR reaction mixtures contained 10 μl of 2X SYBR Green Master Mix (Applied Biosystems, USA), 10 μM of forward and reverse primers, and 1 μl sample cDNA. The sequences of primers used for RT-PCR were 5′-GGGACGCAGACATCGTCATC-3′ and 5′-TCGTCATCGTCGAAATGGGC-3′ for MMP-9; 5′-ATCTGGCACCACACCTTC-3′ and 5′-AGCCAGGTCCAGACGCA-3′ for β-actin. Amplification was performed using the QuantStudio7 system (Applied Biosystems) at the following conditions: 2 min at 50°C, 10 min at 95°C, followed by 45 cycles of 15 s at 95°C, and 1 min at 60°C. Relative gene expression was calculated using the 2^−ΔΔCT^ method.

### Gelatin Zymography

Gelatin zymography was performed according to standard protocols. 6 × 10^5^ MDA-MB-231 cells were seeded overnight in 60 mm culture plates. Cells were treated with different concentrations of CME (0, 2.5, 5, 7.5, or 10 μg/ml) for 48 h, and the conditioned media were collected, concentrations equalized, and subjected to gel electrophoresis on Novex 10% Zymogram Plus gels (Invitrogen, USA) with a fixed concentration of 10% gelatin. After electrophoresis, gelatinase in gels was activated by first incubating in renaturing buffer (Thermo Fisher Scientific, USA) for 30 m, washing with MilliQ water and then incubating in developing buffer (Thermo Fisher Scientific, USA) for another 30 m. Gels were stained with 0.25% Coomassie blue solution and destained in 5% methanol and 10% acetic acid. Enzymatic activity appears as cleared bands on a dark background. Images of gels were taken using a Chemidoc Imaging System (Bio-Rad Laboratories, Hercules, CA, USA) and quantified using Image Lab software (Bio-Rad, USA).

### Western Blot Analysis

MDA-MB-231 cells treated with various concentrations (0, 2.5, 5, 10, or 20 μg/ml) of CME were harvested after 15, 30, 45 m, 1,2, 8, 16, 24, or 48 h. Cell pellets were lysed in RIPA buffer (50 mM Tris-HCl, pH 7.4, 150 mM NaCl, 1 mM ethylenediaminetetraacetic acid (EDTA), 1% Triton X-100, 1% sodium deoxycholate, and 0.1% SDS). Detergent compatible (DC) Protein Assay (Bio-Rad, Hercules, CA, USA) was used to determine protein concentrations. Equal amounts of cell lysates were electrophoresed through sodium dodecyl sulfate-polyacrylamide gel electrophoresis (SDS-PAGE) gels and transferred onto polyvinylidene fluoride (PVDF) membranes (Bio-Rad). The blots were then blocked in 5% non-fat skim milk and probed with diluted primary antibodies overnight: cleaved-poly(ADP-ribose) polymerase (PARP), PARP, cleaved caspase 3, caspase 3, cleaved caspase 9, caspase 9, caspase 8, phospho-IKKα/β, IKKα, IKKβ, phospho-IκBα, IκBα, phospho-NF-κB, NF-κB, phospho-mTOR, mTOR, phospho-AKT, AKT, phospho-STAT3, and STAT3 (Cell Signaling Technology, USA), EGFR (BD Transduction Laboratories, USA), and β-actin (Santa Cruz Biotechnology, USA) as a control. Blots were then incubated with the corresponding goat anti-rabbit or goat anti-mouse (Life Technologies, USA) horseradish peroxidase (HRP)-conjugated secondary antibodies. Protein bands were visualized using Clarity enhanced chemiluminescence (ECL) or Clarity Max Western blotting substrates (Bio-Rad, USA). Images were obtained using a ChemiDoc Imaging System (Bio-Rad, USA) and protein expression was quantified using Image Lab software (Bio-Rad, USA).

### MDA-MB-231 Xenograft Mouse Model

MDA-MB-231 cells were injected subcutaneously (5 × 10^6^ cells/injection, 50:50 v/v medium:matrigel) into the flanks of female BALB/c nude mice. When tumor volumes reached ~100 mm^3^, animals were randomly assigned to treatment groups (*n* = 4 per group). CME (25 mg/kg, low dose or 50 mg/kg, high dose), or an equal volume of vehicle (0.5% CMC) was administered daily via oral gavage. Tumor size, body weight, food, and water consumption were measured three times a week. Tumor sizes were calculated using the formula, volume = (length × width^2^)/2 mm^3^. At the end of the 28-days treatment period, mice were sacrificed, and tumors and vital organs excised for further analysis. All animal experiments were approved by the Hong Kong Polytechnic University Animal Subjects Ethics Sub-committee and conducted in accordance with the Institutional Guidelines and Animal Ordinance of the Department of Health.

### Statistical Analyses

Statistical analyses were performed using one-way ANOVA with the Least Significant Difference (LSD) *post-hoc* test. Data are presented as mean ± standard deviation (*SD*) or standard error of the mean (SEM) of three independent experiments. ^*^P < 0.05, ^**^P < 0.01, and ^***^P < 0.001 were considered as significant differences.

## Results

### CME Exhibited Cytotoxicity in Breast Cancer Cell Lines

**Figure 1:**
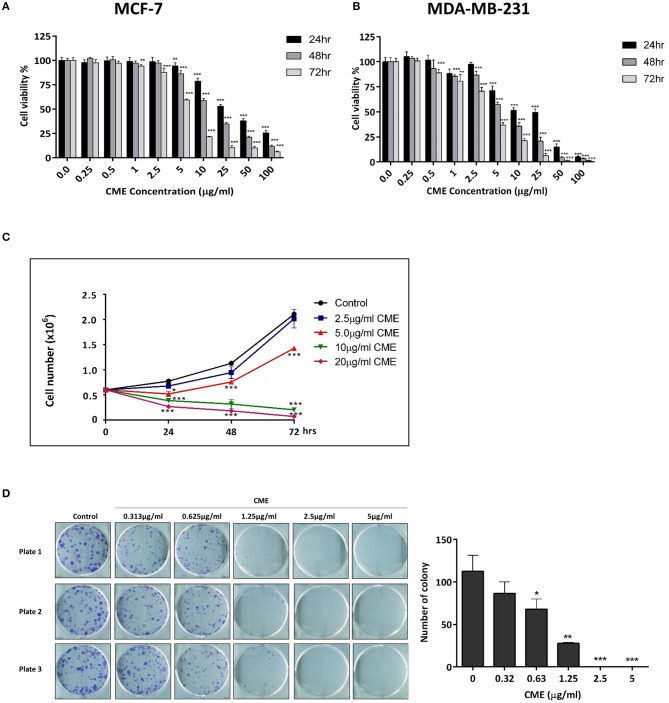
CME time- and dose-dependently reduced cell viability, inhibited cell proliferation, and colony formation in breast cancer cells. Cell viability assay of **(A)** MCF-7 and **(B)** MDA-MB-231 cells. Cells were treated with CME at the indicated concentrations for 24, 48, and 72 h. Cell viability was measured by MTT assay. **(C)** Cell proliferation assay of CME in MDA-MB-231 cells. Cells were treated with CME at the indicated concentrations for 24, 48, and 72 h, after which viable cells were counted. **(D)** Clonogenic assay showing colony formation of MDA-MB-231 cells treated with the indicated concentrations of CME for 14-days. Data are presented as the means ± *SD* from three independent experiments. *P < 0.05, **P < 0.01, and ***P < 0.001 vs. control.

To assess the direct cytotoxicity of CME in breast cancer cells, MCF-7, and MDA-MB-231 were treated with CME and analyzed using the MTT assay. We found that CME exerted dose- and time-dependent cytotoxic effects on both cell lines. In MCF-7 cells, the half inhibitory concentrations (IC_50_) of CME were 27.17, 8.90, and 5.23 μg/ml for 24, 48, and 72 h treatments, respectively (Figure 1A). The IC_50_ values of CME in MDA-MB-231 were 19.96, 12.01, and 5.55 μg/ml, respectively for 24, 48, and 72 h treatments (Figure 1B). Notably, the inhibitory effect of CME in MDA-MB-231 was comparable to or greater than that in MCF-7, indicating that CME may be an effective cytotoxic agent against TNBC.

### CME Inhibited TNBC Cell Proliferation and Colony Formation

To further confirm the inhibitory effect of CME on TNBC, cell proliferation, and colony formation assays were performed. MDA-MB-231 cells were treated with various doses of CME and the number of viable cells were counted at 24, 48, and 72 h after treatment. Results showed that after CME-treatment, viable cell counts dropped significantly, in a dose- and time-dependent manner. At doses of CME >5 μg/ml, a declining trend was seen in the number of viable cells, with a greater effect seen with longer treatment durations (Figure 1C). To evaluate the long-term inhibitory effects of CME in TNBC cells, a 14-days colony formation assay was carried out. We found that CME could significantly reduce the clonogenic ability of MDA-MB-231 at non-toxic concentrations, from doses as low as 0.313 μg/ml. In addition, no colonies were formed at doses higher than 1.25 μg/ml (Figure 1D).

### CME Induced Apoptosis in TNBC Cells

**Figure 2:**
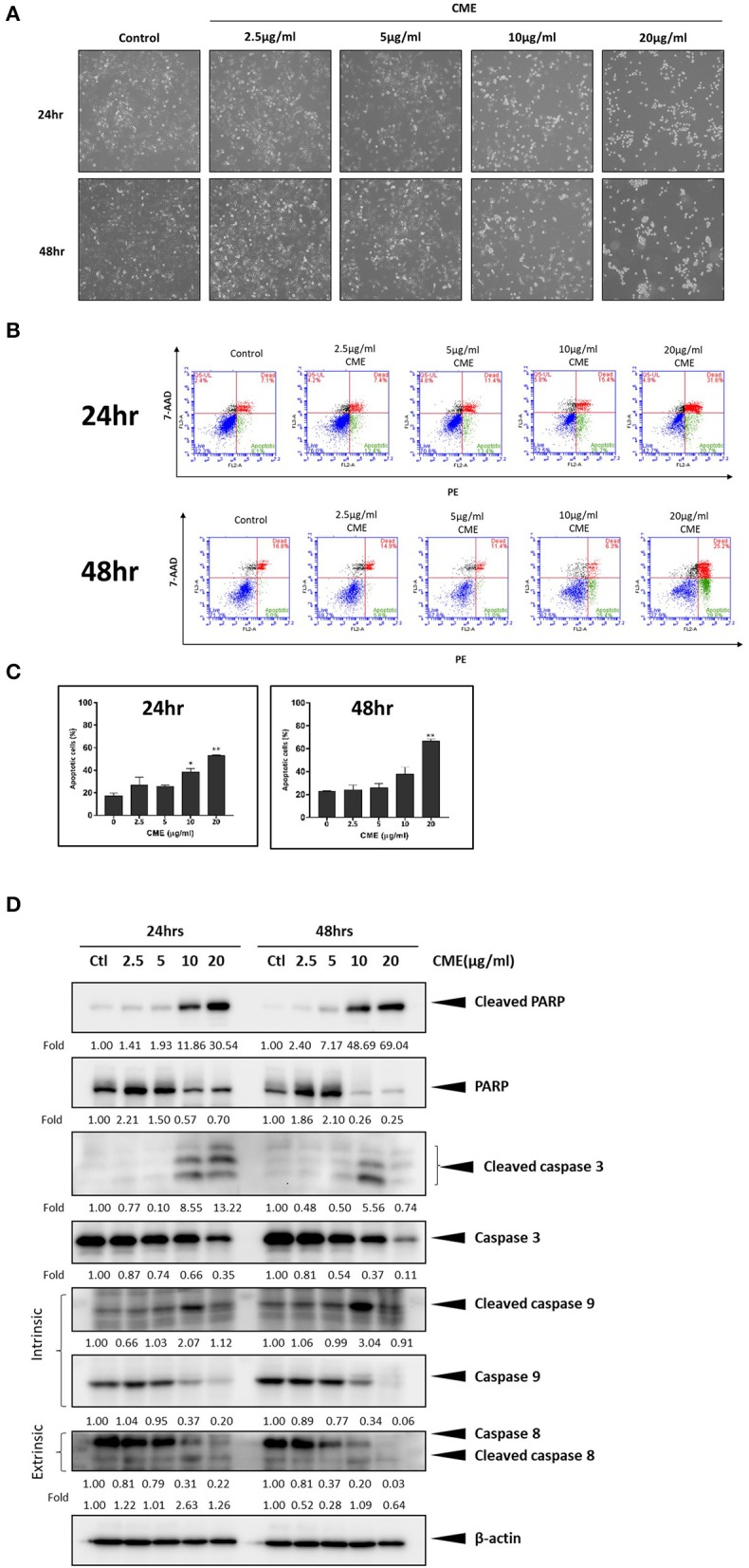
CME induced apoptosis in MDA-MB-231 cells. Cells were treated with CME at the indicated concentrations for 24 and 48 h. **(A)** Morphology of MDA-MB-231 cells was dramatically altered after 24 h of treatment with CME. **(B)** Flow cytometry analysis of apoptosis in annexin V stained, CME-treated MDA-MB-231 cells. **(C)** Proportion of annexin V-positive MDA-MB-231 cells. **(D)** Western blot analysis of apoptosis related proteins. β-Actin was probed as the loading control. Data are presented as the means ± *SD* from three independent experiments. *P < 0.05 and **P < 0.01 vs. control.

Treatment with CME induced morphological changes in MDA-MB-231 cells, in a dose-dependent manner (Figure 2A). Cells shrank from a rigid and spindle-like shape to a rounded shape starting at 10 μg/ml CME, and at 20 μg/ml almost all cells exhibited a rounded morphology. As cell shrinkage and rounding is a characteristic morphological change of cells undergoing apoptosis, we then examined if CME could induce apoptosis in TNBC cells. MDA-MB-231 cells were treated with various concentrations of CME and then assessed via annexin V-PE/7-AAD flow cytometric analysis. Our results showed that CME could significantly increase the proportion of apoptotic (annexin V-positive) cells, in a dose- and time-dependent manner (Figures 2B,C). At 48 h, the percentage of apoptotic cells increased slightly from a baseline of 3–5% with 5 μg/ml CME treatment, and then rose sharply to 15 and 44% with 10 and 20 μg/ml CME treatment, respectively. Western blotting results showed that CME could effectively induce cleavage of PARP, caspase-9, 7, and 3, and also downregulate the expression of total forms of PARP and the aforementioned caspases, in a dose-dependent manner (Figure 2D). These results indicated that CME could indeed induce apoptosis in TNBC cells.

### CME Inhibited Cell Migration in TNBC Cells

**Figure 3:**
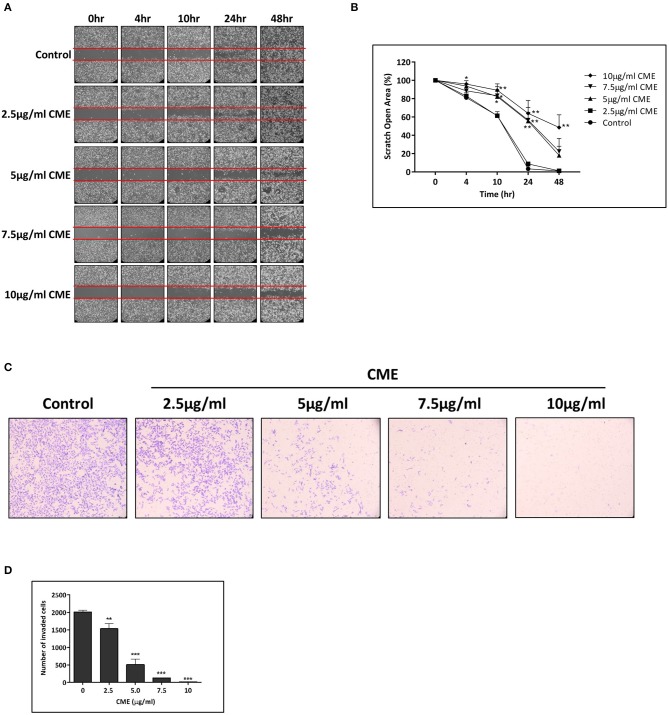
CME inhibited cell migration and invasion in MDA-MB-231. MDA-MB-231 cells were treated with various concentrations of CME (2.5, 5, 7.5, and 10 μg/ml) for the indicated durations. **(A,B)** Cell migration was assessed via wound-healing assay and quantified. **(C,D)** Cell invasion was assessed by the transwell invasion assay, and the number of invasive cells quantified. Data are presented as the means ± *SD* from three independent experiments. *P < 0.05, **P < 0.01, and ***P < 0.001 vs. control.

TNBC possesses an increased potential for invasion and metastasis compared to other types of breast cancers and cell motility is a key indicator of the metastatic capability of cancer cells. Therefore, we assessed the inhibitory effect of CME on the motility of MDA-MB-231 cells using a wound healing assay. Scratch wounds of a fixed size were formed in confluent monolayers of cells, and then cells were incubated in the presence of various concentrations of CME for 48 h. Cells migrated to the cleared zone and the scratch open area was analyzed. Results showed that CME could suppress MDA-MB-231 cell migration in a dose-dependent manner. At 24 h, treatment with CME (5, 7.5, and 10 μg/ml) inhibited cell migration by 55.5, 56.5, and 63.9%, respectively. After 48 h, trends in the inhibitory effect of CME were maintained, with an 18.1, 22.4, and 48.6% scratch wound open area after treatment with 5, 7.5, and 10 μg/ml CME, respectively, while wounds in the control group were completely closed (Figures 3A,B).

### CME Inhibited Cell Invasion in TNBC Cells

To further examine the effect of CME on the invasive capacity of treated cells, a transwell invasion assay was employed. Our results demonstrated that the invasion of MDA-MB-231 cells into the lower chamber was significantly reduced by CME, and a 23.7, 74.6, 93.4, and 98.9% reduction in cell invasion was observed after 48 h of treatment with 2.5, 5, 7.5, and 10 μg/ml CME, respectively (Figures 3C,D). These results suggest that CME could effectively inhibit the metastatic potential of TNBC, *in vitro*.

### CME Downregulated the Protein Expression Levels and Enzymatic Activity of MMP-9

**Figure 4:**
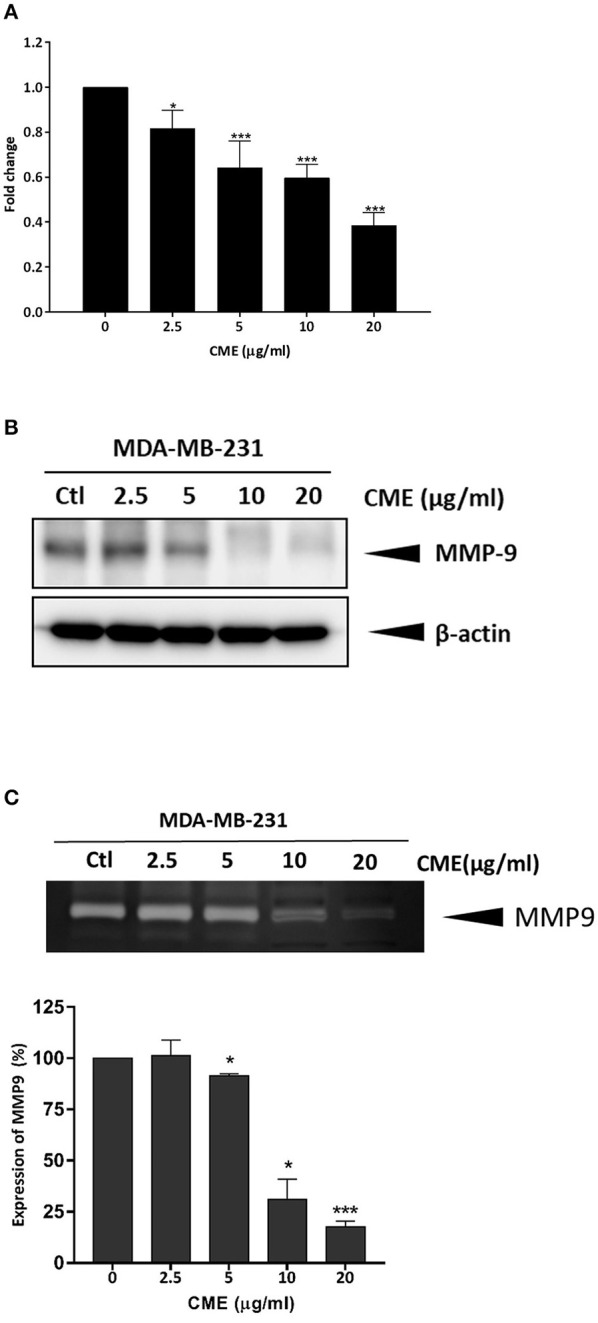
Effect of CME on the expression and activity of MMP-9 in MDA-MB-231 cells. Cells were treated with various concentrations (0, 2.5, 5, 10, and 20 μg/ml) of CME for 48 h. Cell pellets and the conditioned serum-free medium were collected. **(A)** qPCR assessment of the mRNA expression of MMP-9. **(B)** Western blot assessment of the protein expression of MMP-9. **(C)** Gelatin zymography was employed to analyze the activity of MMP-9. Activity of MMP-9 was quantified by densitometric analysis. Results are presented as the means ± *SD* from three independent experiments. *P < 0.05 and ***P < 0.001 vs. control.

MMPs play critical functions in cancer progression and in particular, MMP-9 has been shown to play a major role in cancer cell migration and invasion (28). Thus, we investigated the effect of CME on the protein expression of MMP-9, and its activity. qPCR analysis identified a dose-dependent inhibition of MMP-9 gene expression and protein expression by CME (Figures 4A,B). Further, using a zymographic method, we showed that CME treatment could reduce the gelatinase activity of MMP-9 in a dose-dependent manner (Figure 4C).

### CME Downregulated Expression of EGFR in TNBC Cells

**Figure 5:**
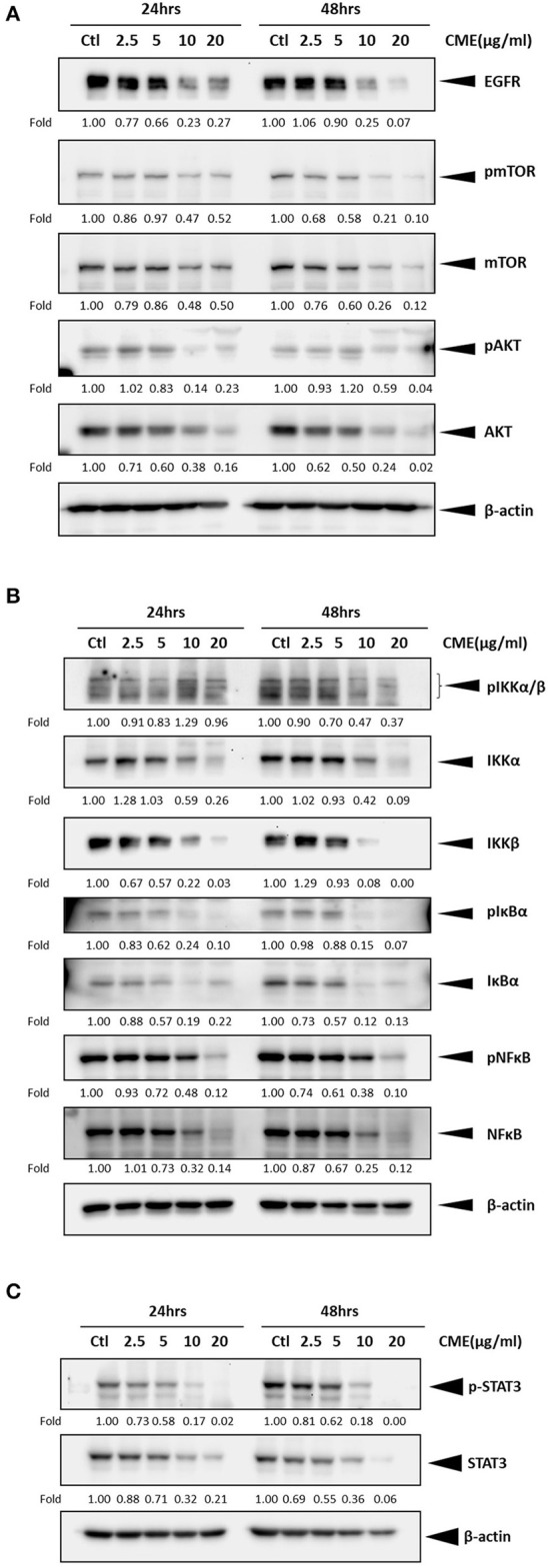
CME suppressed NF-κB, STAT3, and PI3K/AKT/mTOR signaling in MDA-MB-231 cells. MDA-MB-231 cells were treated with CME at the indicated concentrations for 24 or 48 h. Cell pellets were collected for Western blot analysis. The expression of key proteins in the **(A)** PI3K/AKT/mTOR, **(B)** NF-κB, and **(C)** STAT3 signaling pathways. β-Actin was probed as a loading control. Protein expression is indicated, as fold of control. Three independent experiments were conducted and representative blots are shown.

**Figure 6:**
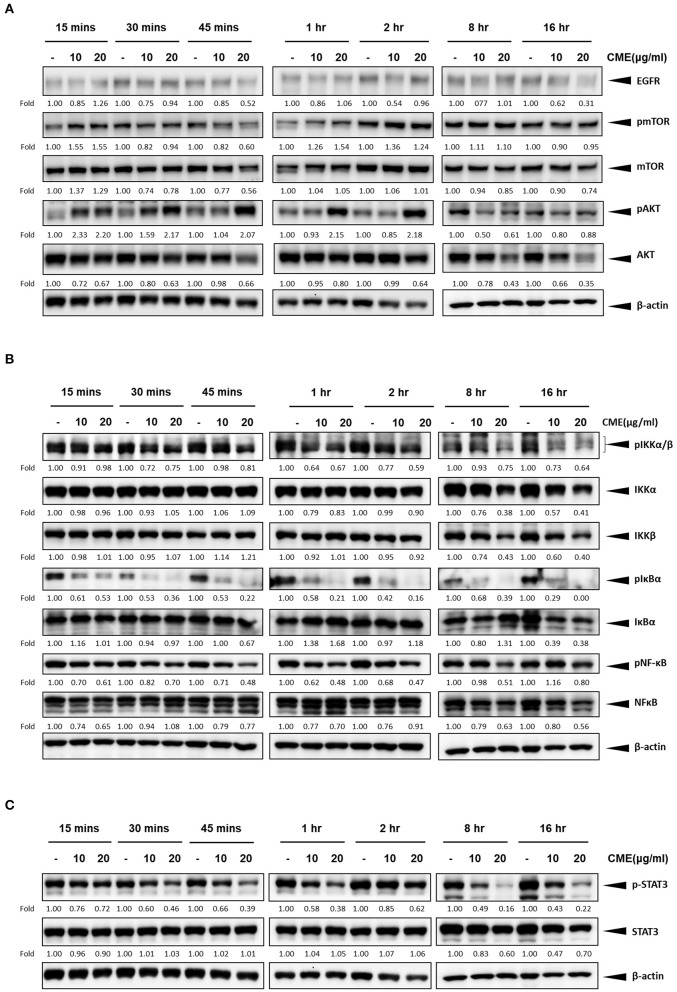
CME suppressed NF-κB, STAT3, and PI3K/AKT/mTOR signaling in MDA-MB-231 cells. MDA-MB-231 cells were treated with CME at 10 or 20 μg/ml for the indicated time points, from 15 min to 16 h. Cell pellets were collected for Western blot analysis. The expression of key proteins in the **(A)** PI3K/AKT/mTOR, **(B)** NF-κB, and **(C)** STAT3 signaling pathways. β-Actin was probed as a loading control. Protein expression is indicated, as fold of control. Three independent experiments were conducted and representative blots are shown.

As previous reports have shown that epidermal growth factor receptor (EGFR) expression is upregulated in TNBC and could be a potential therapeutic target (29, 30), we investigated if CME could mediate the protein expression of EGFR. Western blot results showed that CME could significantly downregulate the protein expression of EGFR, in a dose- and time-dependent manner (Figures 5A, 6A), providing some initial insight into its potential mechanistic activity. Further, as studies have demonstrated that EGFR could affect numerous downstream carcinogenic pathways, including the AKT/mTOR, NF-κB, and STAT3 pathways (31–34), we next investigated the effects of CME on expression of key players in these pathways.

### CME Downregulated PI3K/AKT/mTOR Signaling in TNBC Cells

The PI3K/AKT/mTOR pathway is one of the most important cancer-promoting pathways, and affects a host of key factors that can trigger a series of events favoring cancer cell growth and metastasis, including activation of NF-κB and MMP-9 (35). Therefore, we also examined if CME could downregulate key proteins of PI3K/AKT/mTOR signaling pathway in MBA-MB-231 cells. Our Western blot results confirmed that CME could significantly reduce the protein expression of phospho-mTOR and AKT in a dose- and time-dependent manner (Figures 5A, 6A). These results further demonstrated that CME could inhibit important pro-cancer pathways in TNBC.

### CME Downregulated NF-κB Signaling in TNBC Cells

Previous studies have shown that the NF-κB signaling pathway is an important player in the development and progression of breast cancer (36). Therefore, we investigated whether CME could suppress NF-κB signaling. Our results showed that treatment of MDA-MB-231 cells with CME could dose- and time-dependently decrease the protein expression of key markers in the NF-κB signaling pathway, including phospho-NF-κB, IκBα, and IKKα/β. These findings indicated that CME-induced anti-cancer activity may potentially occur by blocking the NF-κB signaling pathway (Figures 5B, 6B).

### CME Downregulated STAT3 Signaling in TNBC Cells

It has been demonstrated that aberrant STAT3 activation in cancer plays a key role in invasion and metastasis (37). In addition, researchers have found that ~70% of breast cancers exhibit constitutive activation of STAT3, and STAT3 has been strongly associated with TNBC (32, 38). Therefore, we investigated whether CME treatment could downregulate the STAT3 signaling pathway. Our results demonstrated that CME could significantly downregulate the expression of phospho-STAT3 in a dose- and time-dependent manner (Figures 5C, 6C), suggesting that the anti-metastatic activity of CME in TNBC cells may potentially involve the downregulation of STAT3 signaling.

### CME Inhibited Tumor Growth in the MDA-MB-231 Subcutaneous Tumor Xenograft Mouse Model

**Figure 7:**
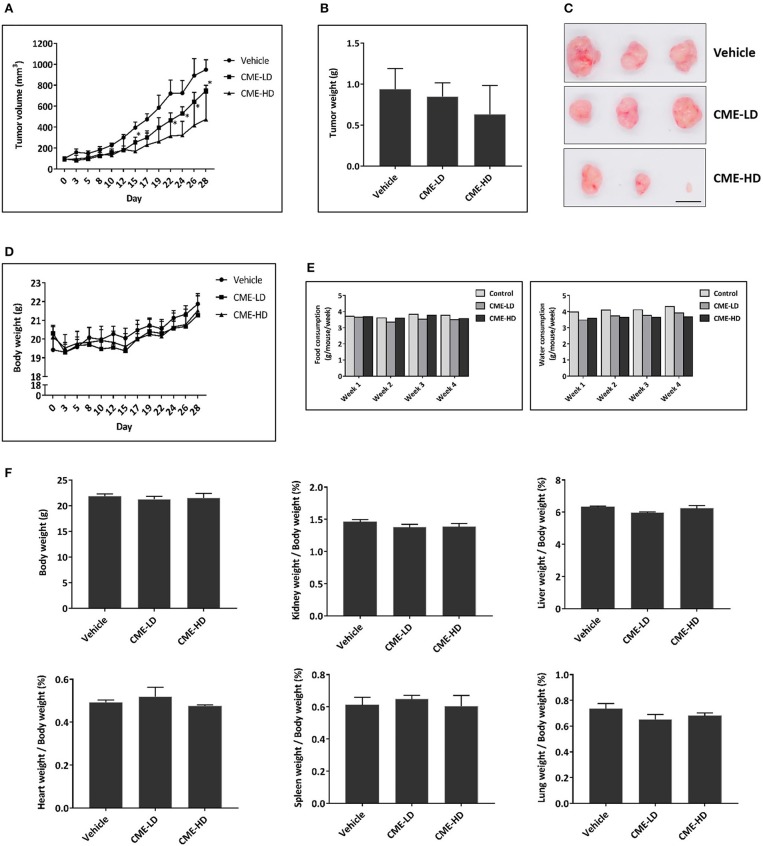
CME inhibited the growth of TNBC *in vivo*. A xenograft breast cancer model was established by subcutaneous injection of MDA-MB-231 cells into BALB/c nude mice. Upon development of tumors to a palpable size, mice were randomized into different groups and received daily treatment with vehicle (0.5% CMC), CME-LD (25 mg/kg), or CME-HD (50 mg/kg) via oral gavage. The experimental period lasted for 28-days, after which mice were sacrificed, and their tumors and organs excised for further analysis. **(A)** Longitudinal assessment of tumor volume. **(B)** Tumor weight at experimental endpoint. **(C)** Representative photographs of tumors at experimental endpoint. Scale bar, 10 mm. **(D)** Mouse body weight, and **(E)** food and water consumption throughout the 28-days experimental period. **(F)** Mean organ weights normalized to body weights. Error bars represent means ± SEM. *P < 0.05 compared to control.

To investigate the anti-cancer effect of CME *in vivo*, we subjected MDA-MB-231 xenograft mice to daily treatment with CME at low (25 mg/kg) or high (50 mg/kg) doses. These doses were selected based on conversion of the human dose of 0.1–0.2 g/day (~2–4 mg/kg in a human of 50 kg) to the mouse equivalent dose, using the body surface area normalization method as detailed previously (39). After 4 weeks treatment, mice were euthanized, and tumors and organs were removed for assessment. Compared to vehicle control, CME treatment significantly and dose dependently suppressed tumor growth. Endpoint tumor weight was also decreased by CME, although this effect did not reach statistical significance. At day 28, average tumor volumes were reduced by 21.1% (CME low dose) and 49.9% (CME high dose) when compared to mice treated with vehicle. Average tumor weights were reduced by 9.6% (CME low dose) and 32.6% (CME high dose) when compared to control (Figures 7A–C). Notably, body weight, food, and water consumption of mice were unaffected by CME treatment (Figures 7D,E), and CME treatment did not cause any significant changes in vital organ weights (Figure 7F). Together, these results suggest that CME possesses *in vivo* anti-cancer efficacy without causing appreciable toxicity.

## Discussion

*Centipeda minima* (L.) A. Braun & Asch. is an annual small herb commonly found in Asia for alleviating headaches, infection, and respiratory discomfort. In previous studies, its extract (CME) has been found to be possess anti-inflammatory (40), anti-angiogenic (41), and anti-cancer (13, 17) effects, through by inhibition of NF-κB and activation of Nrf2 (42), however its effects on TNBC had yet to be investigated.

In our initial results, we showed that CME could exhibit cytotoxicity in both MCF-7 (ER positive) and MDA-MB-231 (triple negative) breast cancer cell lines. However, as the inhibitory effect of CME in MDA-MB-231 was comparable to or greater than that in MCF-7, we focused on the effects of CME in TNBC.

In TNBC, CME could induce apoptotic cell death via activation of both intrinsic and extrinsic apoptosis pathways. CME significantly downregulated cleavage of PARP, caspase 3, caspase 8, and caspase 9, key markers of the extrinsic and intrinsic apoptosis pathways, in a dose- and time-dependent manner. The intrinsic pathway, also referred to as the mitochondrial pathway, is activated by intracellular stress events including DNA damage, deprivation of growth factors, and release of reactive oxygen species (ROS). In the extrinsic pathway, apoptosis is triggered by the binding of extracellular death ligands to their associated death receptors on the cell surface (43). As, previous studies have shown, the co-activation of both intrinsic and extrinsic apoptotic pathways can lead to a synergistic amplification of apoptosis and increased efficacy of cancer cell killing (44).

MMP-9 is a type IV collagenase which is commonly overexpressed and upregulated in the epithelium of cancer cells. The role of MMP-9 in metastasis and invasion in cancers including prostate cancer (45, 46), colorectal cancer (47), and breast cancer (48, 49) is well-known, and previous publications have shown that MMP-9 directly contributes to cancer metastasis and invasion. When MMP-9 is translated and activated, enzymatic activity digests type IV collagen in the extracellular matrix (ECM), which favors cancer cell metastasis to nearby tissues, and eventually invasion to other organs by disrupting local cell basement membranes. CME was also shown to suppress cancer cell migration and invasion, and activity of MMP-9. Suppression of MMP-9 not only inhibits the invasiveness and metastasis of tumor cells, but can also affect angiogenesis and cell growth. MMP-9 expression and activity has been linked to AKT/mTOR, NF-κB, and STAT3 pathways. Previous *in vitro* studies in breast cancer cell lines have shown that PI3K/AKT signaling can activate MMP-9 (50, 51), the MMP-9 promoter contains an NF-κB binding site (52), and siRNA inhibition of STAT3 in breast cancer cells could suppress MMP-9 (53). Thus, inhibition of these pathways by CME could potentially result in the decreased MMP-9 activity and suppression of the metastatic phenotype demonstrated in our results.

While it may be sufficient to target a single entity for single gene disorders, diseases are often the result of a multitude of different factors, both internal and external. Therefore, treatment of complex disorders, such as cancer, may be rendered more effective by addressing multiple targets at once. One major advantage of TCM and herbal medicines over conventional single-entity drugs are that they comprise multiple active ingredients, and can potentially produce additive or synergistic effects by interacting with multiple targets simultaneously (54). Additionally, inhibition of a single oncogene or oncogenic driver may more readily induce development of resistance through compensatory mutations or activation of alternative signaling pathways. Thus, it may be advantageous to employ therapeutic strategies which target multiple pathways at once, and accordingly, such strategies are increasingly finding credence in cancer therapy (55). In our investigation, we found that CME could inhibit multiple signaling pathways, including the PI3K/AKT/mTOR, NF-κB, and STAT3 pathways. Previous studies have implicated these three pathways as important players in TNBC, and they have already been selected as targets in a number of anti-cancer investigations and clinical studies.

The Cancer Genome Atlas has identified PI3K activating mutations as the most common activating mutations in TNBC (present in 10.2% of cases), and in addition, other researchers have found that 9.6% of TNBCs exhibit a loss of PTEN, a negative regulator of the PI3K pathway (56, 57). Supporting the importance of this pathway in TNBC, in another study it was found that ~36% (36/99) of TNBC patients had PI3K pathway-activated tumors (58). Due to the above, PI3K/AKT/mTOR has been identified as a major actionable pathway in TNBC. In a phase II clinical trial, Ipatasertib, a highly selective AKT inhibitor, was tested in combination with paclitaxel as a first-line therapy for metastatic TNBC. In the intention to treat population, ipatasertib improved median progression-free survival (PFS) when compared to placebo (6.2 vs. 4.9 months), and in an subpopulation of patients with PIK3CA/AKT1/PTEN-altered tumors, median PFS was improved in placebo vs. ipataertib, from 4.9 to 9.0 months (59). In another phase II clinical trial, on the efficacy of novel drugs in combination with standard chemotherapy vs. standard therapy alone, paclitaxel with MK-2206, an allosteric AKT inhibitor, improved the pathological complete response (here defined as the absence of invasive cancer in breast and nodes) rate when compared to paclitaxel alone (40.2 vs. 22.4%). The predicted probability of success for MK-2206 in phase III trials was calculated to be 75.9% (60).

NF-κB is commonly found upregulated in breast cancers, and plays key roles in areas including cell survival, proliferation, inflammation, and immunity (61). In a previous study, the NFKBIA gene (encoding the NF-κB inhibitor IκBα) was found to be deleted in 10.8% of breast cancer cases, and NFKBIA deletions were shown to be significantly associated with TNBC (present in 32.8% of cases). In *in vitro* investigations, restoration of NFKBIA expression or pharmacologic NF-κB inhibition was shown to attenuate the malignant phenotype of *NFKBIA*-null TNBC cells (62).

Studies have shown that JAK/STAT signaling can promote tumor cell proliferation, survival, invasiveness, and metastasis, and also inhibit the anti-tumor immune response (63). Furthermore, the IL-6/JAK/STAT3 pathway has been shown to be important for the proliferation of CD44^+^CD24^−^ stem cell–like breast cancer cells. These cells are enriched in basal-like tumors, which share a large overlap with TNBC (A study by Bertucci et al. (64) found that 71% of TNBCs could be categorized as basal-like, while 77% of basal-like cancers could be classified as TNBC). Additionally, in a study of patients with metastatic TNBC, IHC staining of tumors showed that 40.4% of patients possessed moderate- or high-levels of pSTAT3 (65). In another study, treatment with a pan-JAK inhibitor was shown to inhibit the viability of basal-like cell lines, and a JAK2 inhibitor was shown to reduce tumor growth in murine cell line and primary breast cancer xenograft models (66). In further pre-clinical studies, co-treatment of cells with paclitaxel and a JAK2 inhibitor, NVP-BSK805, could significantly reduce STAT3 phosphorylation, and *in vivo* tumor growth, when compared with paclitaxel alone (67). However, in a phase II trial of metastatic TNBC patients treated with ruxolitinib, a JAK1/2 inhibitor, though on-target inhibition and decreased levels of pSTAT3 were seen, the primary endpoint of objective responses was not met (65).

Taking together the above work demonstrating the value of PI3K/AKT/mTOR, NF-κB, and STAT3 pathway inhibition in breast cancer, and the failure of some single pathway inhibitors in clinical trials, a strong basis is provided to support the simultaneous targeting of multiple pathways by CME for the treatment of TNBC.

In addition, in our *in vivo* experiments in the TNBC subcutaneous xenograft model, we found that at high doses, CME significantly and effectively reduced tumor volumes by ~50%. Treatment with commonly used chemotherapeutics can often lead to significant side effects, for example, treatment with cisplatin led to body weight losses of up 10% in TNBC xenograft mice (68), whereas in our study, treatment with the herbal extract CME did not affect body weight or vital organs. Thus our *in vivo* results provide additional support for CME as an effective and non-toxic therapeutic for TNBC.

## Conclusion

**Figure 8:**
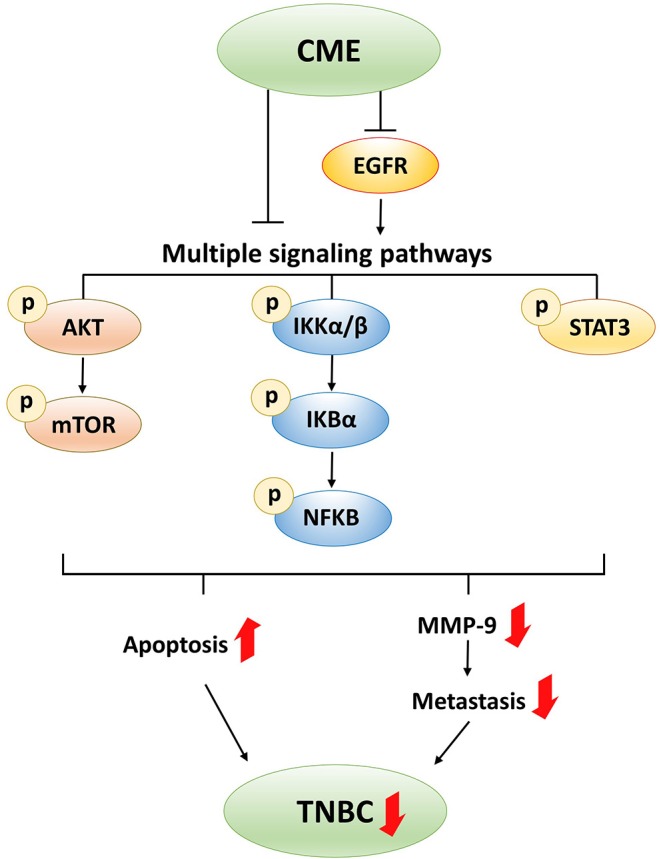
Proposed anti-cancer mechanism of CME in TNBC.

In this study, we showed that CME could induce intrinsic and extrinsic pathways of apoptotic cell death, and suppress MMP-9 and metastasis in the MDA-MB-231 TNBC breast cancer cell line. These anti-cancer effects may potentially result from inhibition of EGFR and downregulation of the PI3K/AKT/mTOR, NF-κB, and STAT3 signaling pathways (Figure 8). The *in vivo* anti-cancer efficacy of CME was also investigated, and it was shown to exhibit anti-tumorigenic effects, without inducing toxicity. These findings provide strong evidence for the potential development of CME as a novel therapeutic agent with multi-pathway-inhibitory activity and high efficacy against TNBC.

## Data Availability Statement

The original contributions presented in the study are included in the article, further inquiries can be directed to the corresponding author.

## Ethics Statement

All animal experiments were approved by the Hong Kong Polytechnic University Animal Subjects Ethics Sub-committee and conducted in accordance with the Institutional Guidelines and Animal Ordinance of the Department of Health.

## Author Contributions

WT, SC, and DM contributed to the conceptualization and design of the study. ML, BC, W-YW, ZQ, M-SC, T-WL, and YL conducted the experiments. ML and BC wrote the manuscript. WT, SC, and BC contributed to manuscript revision. All authors read and approved the final manuscript.

### Conflict of Interest

The authors declare that the research was conducted in the absence of any commercial or financial relationships that could be construed as a potential conflict of interest.

## References

[1] Bray F Ferlay J Soerjomataram I Siegel RL Torre LA Jemal A. Global cancer statistics 2018: GLOBOCAN estimates of incidence and mortality worldwide for 36 cancers in 185 countries. CA Cancer J Clin. (2018) 68:394–424. 10.3322/caac.21492 30207593

[2] Foulkes WD Smith IE Reis-Filho JS. Triple-negative breast cancer. N Engl J Med. (2010) 363:1938–48. 10.1056/NEJMra1001389 21067385

[3] Garrido-Castro AC Lin NU Polyak K. Insights into molecular classifications of triple-negative breast cancer: improving patient selection for treatment. Cancer Discov. (2019) 9:176–98. 10.1158/2159-8290.CD-18-1177 30679171 PMC6387871

[4] Dent R Trudeau M Pritchard KI Hanna WM Kahn HK Sawka CA. Triple-negative breast cancer: clinical features and patterns of recurrence. Clin Cancer Res. (2007) 13(15 Pt 1):4429–34. 10.1158/1078-0432.CCR-06-3045 17671126

[5] Bianchini G Balko JM Mayer IA Sanders ME Gianni L. Triple-negative breast cancer: challenges and opportunities of a heterogeneous disease. Nat Rev Clin Oncol. (2016) 13:674–90. 10.1038/nrclinonc.2016.66 27184417 PMC5461122

[6] Abramson VG Lehmann BD Ballinger TJ Pietenpol JA. Subtyping of triple-negative breast cancer: implications for therapy. Cancer. (2015) 121:8–16. 10.1002/cncr.28914 25043972 PMC4270831

[7] Carey LA Dees EC Sawyer L Gatti L Moore DT Collichio F. The triple negative paradox: primary tumor chemosensitivity of breast cancer subtypes. Clin Cancer Res. (2007) 13:2329–34. 10.1158/1078-0432.CCR-06-1109 17438091

[8] Andre F Zielinski CC. Optimal strategies for the treatment of metastatic triple-negative breast cancer with currently approved agents. Ann Oncol. (2012) 23(Suppl 6):vi46–51. 10.1093/annonc/mds195 23012302

[9] Yuan H Ma Q Ye L Piao G. The traditional medicine and modern medicine from natural products. Molecules. (2016) 21:559. 10.3390/molecules21050559 27136524 PMC6273146

[10] Ngo LT Okogun JI Folk WR. 21st century natural product research and drug development and traditional medicines. Nat Prod Rep. (2013) 30:584–92. 10.1039/c3np20120a 23450245 PMC3652390

[11] Tang W Eisenbrand G *Centipeda minima* (L.) A. Braun et Aschers. Chinese Drugs of Plant Origin. Berlin, Heidelberg: Springer Berlin Heidelberg (1992). pp. 277–9.

[12] Wu P Su M-X Wang Y Wang G-C Ye W-C Chung H-Y. Supercritical fluid extraction assisted isolation of sesquiterpene lactones with antiproliferative effects from *Centipeda minima*. Phytochemistry. (2012) 76:133–40. 10.1016/j.phytochem.2012.01.003 22277736

[13] Liu R Qu Z Lin Y Lee CS Tai WC Chen S. Brevilin A Induces cell cycle arrest and apoptosis in nasopharyngeal carcinoma. Front Pharmacol. (2019) 10:594. 10.3389/fphar.2019.00594 31178739 PMC6544084

[14] Liu R Chan BD Mok DKW Lee CS Tai WCS Chen SB. Arnicolide D, from the herb *Centipeda minima*, is a therapeutic candidate against nasopharyngeal carcinoma. Molecules. (2019) 24:1908. 10.3390/molecules24101908 31108969 PMC6571971

[15] Su T Wang Y-P Wang X-N Li C-Y Zhu P-L Huang Y-M. The JAK2/STAT3 pathway is involved in the anti-melanoma effects of brevilin A. Life Sci. (2020) 241:117169. 10.1016/j.lfs.2019.117169 31843524

[16] Zhu P Zheng Z Fu X Li J Yin C Chou J. Arnicolide D exerts anti-melanoma effects and inhibits the NF-κB pathway. Phytomedicine. (2019) 64:153065. 10.1016/j.phymed.2019.153065 31408803

[17] Huang SS Chiu CS Lin TH Lee MM Lee CY Chang SJ. Antioxidant and anti-inflammatory activities of aqueous extract of *Centipeda minima*. J Ethnopharmacol. (2013) 147:395–405. 10.1016/j.jep.2013.03.025 23506988

[18] Taylor RS Towers GH. Antibacterial constituents of the Nepalese medicinal herb, *Centipeda minima*. Phytochemistry. (1998) 47:631–4. 10.1016/S0031-9422(97)00534-7 9461679

[19] Liang H Bao F Dong X Tan R Zhang C Lu Q. Antibacterial thymol derivatives isolated from *Centipeda minima*. Molecules. (2007) 12:1606–13. 10.3390/12081606 17960076 PMC6149160

[20] Wu JB Chun YT Ebizuka Y Sankawa U. Biologically active constituents of *Centipeda minima*: sesquiterpenes of potential anti-allergy activity. Chem Pharm Bull (Tokyo). (1991) 39:3272–5. 10.1248/cpb.39.3272 1726075

[21] Su M Wu P Li Y Chung HY. Antiproliferative effects of volatile oils from *Centipeda minima* on human nasopharyngeal cancer CNE cells. Nat Prod Commun. (2010) 5:151–6. 10.1177/1934578X1000500135 20184042

[22] Guo YQ Sun HY Chan CO Liu BB Wu JH Chan SW. *Centipeda minima* (Ebushicao) extract inhibits PI3K-Akt-mTOR signaling in nasopharyngeal carcinoma CNE-1 cells. Chin Med. (2015) 10:26. 10.1186/s13020-015-0058-5 26388933 PMC4575463

[23] Changlong L Hezhen W Yongping H Yanfang Y Yanwen L Jianwen L. 6-O-Angeloylenolin induces apoptosis through a mitochondrial/caspase and NF-kappaB pathway in human leukemia HL60 cells. Biomed Pharmacother. (2008) 62:401–9. 10.1016/j.biopha.2007.10.010 18077129

[24] Wang J Li M Cui X Lv D Jin L Khan M. Brevilin A promotes oxidative stress and induces mitochondrial apoptosis in U87 glioblastoma cells. Onco Targets Ther. (2018) 11:7031–40. 10.2147/OTT.S179730 30410360 PMC6198872

[25] Wang Y Yu RY Zhang J Zhang WX Huang ZH Hu HF. Inhibition of Nrf2 enhances the anticancer effect of 6-O-angeloylenolin in lung adenocarcinoma. Biochem Pharmacol. (2017) 129:43–53. 10.1016/j.bcp.2017.01.006 28104435

[26] You P Wu H Deng M Peng J Li F Yang Y. Brevilin A induces apoptosis and autophagy of colon adenocarcinoma cell CT26 via mitochondrial pathway and PI3K/AKT/mTOR inactivation. Biomed Pharmacother. (2018) 98:619–25. 10.1016/j.biopha.2017.12.057 29289836

[27] Chan CO Jin DP Dong NP Chen SB Mok DK. Qualitative and quantitative analysis of chemical constituents of *Centipeda minima* by HPLC-QTOF-MS & HPLC-DAD. J Pharm Biomed Anal. (2016) 125:400–7. 10.1016/j.jpba.2016.04.029 27131150

[28] Mehner C Hockla A Miller E Ran S Radisky DC Radisky ES. Tumor cell-produced matrix metalloproteinase 9 (MMP-9) drives malignant progression and metastasis of basal-like triple negative breast cancer. Oncotarget. (2014) 5:2736–49. 10.18632/oncotarget.1932 24811362 PMC4058041

[29] Costa R Shah AN Santa-Maria CA Cruz MR Mahalingam D Carneiro BA. Targeting epidermal growth factor receptor in triple negative breast cancer: new discoveries and practical insights for drug development. Cancer Treat Rev. (2017) 53:111–9. 10.1016/j.ctrv.2016.12.010 28104566

[30] Shao F Sun H Deng CX. Potential therapeutic targets of triple-negative breast cancer based on its intrinsic subtype. Oncotarget. (2017) 8:73329–44. 10.18632/oncotarget.20274 29069872 PMC5641215

[31] Xia Y Shen S Verma IM. NF-kappaB, an active player in human cancers. Cancer Immunol Res. (2014) 2:823–30. 10.1158/2326-6066.CIR-14-0112 25187272 PMC4155602

[32] Qin JJ Yan L Zhang J Zhang WD. STAT3 as a potential therapeutic target in triple negative breast cancer: a systematic review. J Exp Clin Cancer Res. (2019) 38:195. 10.1186/s13046-019-1206-z 31088482 PMC6518732

[33] Shostak K Chariot A. EGFR and NF-kappaB: partners in cancer. Trends Mol Med. (2015) 21:385–93. 10.1016/j.molmed.2015.04.001 25979753

[34] Toss A Cristofanilli M. Molecular characterization and targeted therapeutic approaches in breast cancer. Breast Cancer Res. (2015) 17:60. 10.1186/s13058-015-0560-9 25902832 PMC4407294

[35] Porta C Paglino C Mosca A. Targeting PI3K/Akt/mTOR signaling in cancer. Front Oncol. (2014) 4:64. 10.3389/fonc.2014.00064 24782981 PMC3995050

[36] Wang W Nag SA Zhang R. Targeting the NFkappaB signaling pathways for breast cancer prevention and therapy. Curr Med Chem. (2015) 22:264–89. 10.2174/0929867321666141106124315 25386819 PMC6690202

[37] Teng Y Ross JL Cowell JK. The involvement of JAK-STAT3 in cell motility, invasion, and metastasis. JAKSTAT. (2014) 3:e28086. 10.4161/jkst.28086 24778926 PMC3995737

[38] Walker SR Nelson EA Zou L Chaudhury M Signoretti S Richardson A. Reciprocal effects of STAT5 and STAT3 in breast cancer. Mol Cancer Res. (2009) 7:966–76. 10.1158/1541-7786.MCR-08-0238 19491198

[39] Reagan-Shaw S Nihal M Ahmad N. Dose translation from animal to human studies revisited. FASEB J. (2008) 22:659–61. 10.1096/fj.07-9574LSF 17942826

[40] Sarkar A Tripathi V Sahu R Anti-inflammatory and anti-arthritis activity of flavonoids fractions isolated from *Centipeda minima* leaves extracts in rats. Clin Exp Pharmacol. (2017) 7:1–8. 10.4172/2161-1459.1000231

[41] Huang W Yu X Liang N Ge W Kwok HF Lau CB. Anti-angiogenic activity and mechanism of sesquiterpene lactones from *Centipeda minima*. Nat Prod Commun. (2016) 11:435–8. 10.1177/1934578X1601100402 27396185

[42] Li Y Zeng Y Huang Q Wen S Wei Y Chen Y. Helenalin from *Centipeda minima* ameliorates acute hepatic injury by protecting mitochondria function, activating Nrf2 pathway and inhibiting NF-kappaB activation. Biomed Pharmacother. (2019) 119:109435. 10.1016/j.biopha.2019.109435 31520915

[43] Baig S Seevasant I Mohamad J Mukheem A Huri HZ Kamarul T. Potential of apoptotic pathway-targeted cancer therapeutic research: where do we stand? Cell Death Dis. (2016) 7:e2058. 10.1038/cddis.2015.275 26775709 PMC4816162

[44] Schneider-Jakob S Corazza N Badmann A Sidler D Stuber-Roos R Keogh A. Synergistic induction of cell death in liver tumor cells by TRAIL and chemotherapeutic drugs via the BH3-only proteins Bim and Bid. Cell Death Dis. (2010) 1:e86. 10.1038/cddis.2010.66 21368859 PMC3035907

[45] Hwang E-S Park K-K. Magnolol suppresses metastasis via inhibition of invasion, migration, and matrix metalloproteinase-2/-9 activities in PC-3 human prostate carcinoma cells. Biosci Biotechnol Biochem. (2010) 74:961–7. 10.1271/bbb.90785 20460721

[46] Liu K-C Huang A-C Wu P-P Lin H-Y Chueh F-S Yang J-S. Gallic acid suppresses the migration and invasion of PC-3 human prostate cancer cells via inhibition of matrix metalloproteinase-2 and-9 signaling pathways. Oncol Rep. (2011) 26:177–84. 10.3892/or.2011.1264 21503582

[47] Suboj P Babykutty S Gopi DRV Nair RS Srinivas P Gopala S. Aloe emodin inhibits colon cancer cell migration/angiogenesis by downregulating MMP-2/9, RhoB and VEGF via reduced DNA binding activity of NF-κB. Eur J Pharm Sci. (2012) 45:581–91. 10.1016/j.ejps.2011.12.012 22227305

[48] Chen P Lu N Ling Y Chen Y Hui H Lu Z. Inhibitory effects of wogonin on the invasion of human breast carcinoma cells by downregulating the expression and activity of matrix metalloproteinase-9. Toxicology. (2011) 282:122–8. 10.1016/j.tox.2011.01.018 21295103

[49] Xiang L Gilkes DM Chaturvedi P Luo W Hu H Takano N. Ganetespib blocks HIF-1 activity and inhibits tumor growth, vascularization, stem cell maintenance, invasion, and metastasis in orthotopic mouse models of triple-negative breast cancer. J Mol Med (Berl). (2014) 92:151–64. 10.1007/s00109-013-1102-5 24248265 PMC3946681

[50] Ko HS Lee H-J Kim S-H Lee E-O. Piceatannol suppresses breast cancer cell invasion through the inhibition of MMP-9: involvement of PI3K/AKT and NF-κB pathways. J Agricult Food Chem. (2012) 60:4083–9. 10.1021/jf205171g 22480333

[51] Cho SJ Chae MJ Shin BK Kim HK Kim A. Akt- and MAPK-mediated activation and secretion of MMP-9 into stroma in breast cancer cells upon heregulin treatment. Mol Med Rep. (2008) 1:83–8. 10.3892/mmr.1.1.83 21479382

[52] Yan C Boyd DD. Regulation of matrix metalloproteinase gene expression. J Cell Physiol. (2007) 211:19–26. 10.1002/jcp.20948 17167774

[53] Shen Z Zhu D Liu J Chen J Liu Y Hu C. 27-Hydroxycholesterol induces invasion and migration of breast cancer cells by increasing MMP9 and generating EMT through activation of STAT-3. Environ Toxicol Pharmacol. (2017) 51:1–8. 10.1016/j.etap.2017.02.001 28257824

[54] Wang Y Fan X Qu H Gao X Cheng Y. Strategies and techniques for multi-component drug design from medicinal herbs and traditional Chinese medicine. Curr Top Med Chem. (2012) 12:1356–62. 10.2174/156802612801319034 22690682

[55] O'Reilly MS. Targeting multiple biological pathways as a strategy to improve the treatment of cancer. Clin Cancer Res. (2002) 8:3309–10. 12429615

[56] Cancer Genome Atlas Network Comprehensive molecular portraits of human breast tumours. Nature. (2012) 490:61–70. 10.1038/nature11412 23000897 PMC3465532

[57] Shah SP Roth A Goya R Oloumi A Ha G Zhao Y. The clonal and mutational evolution spectrum of primary triple-negative breast cancers. Nature. (2012) 486:395–9. 10.1038/nature10933 22495314 PMC3863681

[58] Martin M Chan A Dirix L O'Shaughnessy J Hegg R Manikhas A A randomized adaptive phase II/III study of buparlisib, a pan-class I PI3K inhibitor, combined with paclitaxel for the treatment of HER2-advanced breast cancer (BELLE-4). Ann Oncol. (2017) 28:313–20. 10.1093/annonc/mdw562 27803006

[59] Kim SB Dent R Im SA Espie M Blau S Tan AR. Ipatasertib plus paclitaxel versus placebo plus paclitaxel as first-line therapy for metastatic triple-negative breast cancer (LOTUS): a multicentre, randomised, double-blind, placebo-controlled, phase 2 trial. Lancet Oncol. (2017) 18:1360–72. 10.1016/S1470-2045(17)30450-3 28800861 PMC5626630

[60] Tripathy D Chien AJ Hylton N Buxton MB Ewing CA Wallace AM. Adaptively randomized trial of neoadjuvant chemotherapy with or without the Akt inhibitor MK-2206: graduation results from the I-SPY 2 trial. J Clin Oncol. (2015) 33:524. 10.1200/jco.2015.33.15_suppl.52425584001

[61] Shostak K Chariot A. NF-kappaB, stem cells and breast cancer: the links get stronger. Breast Cancer Res. (2011) 13:214. 10.1186/bcr2886 21867572 PMC3236328

[62] Bredel M Kim H Thudi NK Scholtens DM Bonner JA Sikic BI NFKBIA deletion in triple-negative breast cancer. J Clin Oncol. (2013) 31(15 Suppl):1012 10.1200/jco.2013.31.15_suppl.1012

[63] Johnson DE O'Keefe RA Grandis JR. Targeting the IL-6/JAK/STAT3 signalling axis in cancer. Nat Rev Clin Oncol. (2018) 15:234–48. 10.1038/nrclinonc.2018.8 29405201 PMC5858971

[64] Bertucci F Finetti P Cervera N Esterni B Hermitte F Viens P. How basal are triple-negative breast cancers? Int J Cancer. (2008) 123:236–40. 10.1002/ijc.23518 18398844

[65] Stover DG Gil Del Alcazar CR Brock J Guo H Overmoyer B Balko J. Phase II study of ruxolitinib, a selective JAK1/2 inhibitor, in patients with metastatic triple-negative breast cancer. NPJ Breast Cancer. (2018) 4:10. 10.1038/s41523-018-0060-z 29761158 PMC5935675

[66] Marotta LL Almendro V Marusyk A Shipitsin M Schemme J Walker SR The JAK2/STAT3 signaling pathway is required for growth of CD44(+)CD24(-) stem cell-like breast cancer cells in human tumors. J Clin Invest. (2011) 121:2723–35. 10.1172/JCI44745 21633165 PMC3223826

[67] Balko JM Schwarz LJ Luo N Estrada MV Giltnane JM Davila-Gonzalez D. Triple-negative breast cancers with amplification of JAK2 at the 9p24 locus demonstrate JAK2-specific dependence. Sci Transl Med. (2016) 8:334ra53. 10.1126/scitranslmed.aad3001 27075627 PMC5256931

[68] Cui L Her S Dunne M Borst GR De Souza R Bristow RG. Significant radiation enhancement effects by gold nanoparticles in combination with cisplatin in triple negative breast cancer cells and tumor xenografts. Radiat Res. (2017) 187:147–60. 10.1667/RR14578.1 28085639

